# Diagnosis of histoplasmosis by next-generation sequencing of microbial cell-free DNA in Texas

**DOI:** 10.1017/ash.2026.10429

**Published:** 2026-06-01

**Authors:** Fernando H. Centeno, Todd Lasco, Mayar Al Mohajer

**Affiliations:** 1 Department of Medicine, Section of Infectious Diseases, Baylor College of Medicine, Houston, USA; 2 Department of Pathology & Immunology, Baylor College of Medicine, Houston, USA

## Abstract

We examine the impact of microbial cell-free DNA (mcfDNA) NGS for the diagnosis of Histoplasma capsulatuim in a quaternary referral center in Texas. Of eight included patients, five had a change in their antifungal coverage after mcfDNA NGS results with faster turnaround times than antigen testing and cultures.

## Background

Histoplasmosis, caused by *Histoplasma capsulatum*, poses a diagnostic challenge due to its worldwide distribution, its heterogeneous clinical presentation, and the limitations of current diagnostic modalities. The clinical spectrum of *Histoplasma* infections is broad and includes pulmonary and extrapulmonary disease, including cardiac, gastrointestinal, and central nervous system disease. Additionally, individuals may present acutely after infection or with chronic undiagnosed disease. Diagnostic testing for this infection has traditionally relied upon culture and histopathology, both of which can frequently miss acute pulmonary infection with this organism due to limited analytical sensitivity. Antigen and serology diagnostics are more sensitive but miss a non-trivial proportion of cases.^1^ Because of these factors, the diagnosis of histoplasmosis is frequently elusive and appropriate antifungal coverage is often delayed while de-escalation of unnecessary antimicrobials is prolonged.

In the United States, next generation sequencing of microbial cell-free DNA (mcfDNA NGS) is available as an open-ended testing assay from patient sera and can detect hundreds of bacteria, fungi, DNA-based viruses, and parasites. At Baylor-St. Luke’s Medical Center (BSLMC), a quaternary medical center (Houston, Texas), mcfDNA NGS is often used once traditional diagnostics (eg, antigen assays, etc.) were unrevealing or in cases where other tests or specimen sources (eg, tissue biopsy, etc.) were infeasible.

## Methods

The medical records at BSLMC were queried for mcfDNA NGS results with *Histoplasma capsulatum* between 2018 and 2025. Cases were included if they were found to be positive for *Histoplasma capsulatum* mcfDNA NGS. A test was considered positive if the mcfDNA NGS concentration threshold in molecules per microliter (MPM) was above that in a previous validation study of healthy controls.^2^ McfDNA NGS was performed on patient sera using the Karius test (Redwood City, CA) while cultures, cytology, and histopathology were performed in-house. MiraVista Diagnostics (Indianapolis, IN) performed antigen testing.

For cases with positive mcfDNA NGS for *H. capsulatum*, the electronic medical record was reviewed for presenting symptoms, available testing, the first test to establish the diagnosis of histoplasmosis, treatment courses, and outcomes. Clinical turnaround times (TAT) were calculated as the time between collection of serum samples for mcfDNA NGS testing, fungal cultures, tissue biopsy, or bronchoalveolar lavage and the reporting of results. This study was approved by the Baylor Institutional Review Board.

## Results

Eight patients with positive mcfDNA NGS for *H. capsulatum* were included. Their presentation and test results are summarized in table 1. The median age was 57.5 and all but one were male. Three presented with isolated pulmonary disease, two had pulmonary disease in combination with extrapulmonary disease, and three were diagnosed with exclusively extrapulmonary disease. Five had known immunocompromising conditions including HIV and inflammatory bowel disease requiring monoclonal antibody therapy. Two had prosthetic material, including a ventriculoperitoneal shunt and a mechanical mitral valve replacement.

**Table 1.** Clinical characteristics, laboratory testing, and outcomes for eight patients with histoplasmosis Table 1 long description.

Patients	*Histoplasma* antigen, antibody studies (clinical TAT)	Fungal cultures (clinical TAT)	Fungal stain, tissue biopsy or cytology (clinical TAT)	Organisms in mcfDNA NGS and concentration (MPM) (clinical TAT)	Change in management	Disposition and outcome
**27-year-old male with newly diagnosed HIV/AIDS with diarrhea, shock, secondary HLH.**	*Histoplasma* antibody, immunodiffusion: Positive M band (5 days) Urine *Histoplasma* antigen: >25.0 (2 days)	Blood – *H. capsulatum* Bone marrow – *H. capsulatum* (6 days) Skin – *H. capsulatum* (13 days)	Bone marrow biopsy: Budding fungal organisms compatible with *H. capsulatum* (1 day) Skin biopsy: Budding yeast compatible with *H. capsulatum* (5 days)	*H. capsulatum* – detected but not quantified (5 days)	No, diagnosed based on fungal biopsy and started on liposomal amphotericin B	Discharged with itraconazole
**78-year-old male with hypertension presenting with high fever, dyspnea, sputum production, and pancytopenia.**	Urine *Histoplasma* antigen EIA: <0.5 (4 days)	None	None	*H. capsulatum* – 610 MPM *Prevotella melaninogenica* – 57 MPM (3 days)	Yes, started itraconazole	Discharged with itraconazole
**66-year-old male with Crohn’s disease and mechanical aortic valve replacement on weekly adalimumab presenting with chronic weight loss and oral mucosal ulcers. Later re-admitted for 2.5 x 1.5 cm mechanical valve vegetation.**	Urine *Histoplasma* antigen EIA: <0.5 (8 days)	Bronchoalveolar lavage x 2 – no growth Aortic valve – no growth Blood – no growth	Aortic valve biopsy: Fungal organisms compatible with *H. capsulatum* (1 day)	First admission: *H. capsulatum –* 675 (2 days) Second admission: *H. capsulatum –* 2729 (2 days) Following amphotericin: No organisms on mcfDNA NGS (2 days)	Yes, started on itraconazole and later switched to liposomal amphotericin	Discharged with itraconazole following three-week course of liposomal amphotericin B and negative repeat mcfDNA NGS
**55-year-old male with history of ulcerative colitis and intracerebral hemorrhage requiring ventriculoperitoneal shunt presenting with acute-onset encephalopathy.**	Urine *Histoplasma* antigen EIA: <0.5 (3 days) Cerebrospinal fluid *Histoplasma* antigen: <0.4 (6 days)	Cerebrospinal fluid – *H. capsulatum* (6 days)	Bronchoalveolar lavage – Fungal organisms on GMS stain (3 days)	*H. capsulatum* – 586 (2 days)	Yes, liposomal amphotericin B started empirically then switched to itraconazole after a two-week course following mcfDNA NGS results	Transferred to long-term acute care hospital
**20-year-old male who presents with newly diagnosed HIV/AIDS, *Staphylococcus aureus* bacteremia, a diffuse rash, and respiratory failure with reticulonodular pulmonary opacities on imaging.**	Urine *Histoplasma* antigen EIA: >15.0 (3 days)	Bronchoalveolar lavage – *H. capsulatum* (7 days)	Skin biopsy – Numerous small organisms seen on GMS stain compatible with *H. capsulatum* (3 days) Bronchoalveolar lavage – Fungal organisms on GMS stain (1 day)	*H. capsulatum* – detected but not quantified (2 days)	No, started on liposomal amphotericin B based on preceding skin biopsy	Expired from septic shock with multiorgan failure
**36-year-old male with HIV/AIDS and prior disseminated histoplasmosis off therapy presenting with subacute headache, fever, malaise, and dyspnea.**	Urine *Histoplasma* antigen EIA: >15.0 (5 days)	Cerebrospinal fluid – no growth	Bronchoalveolar lavage – oropharyngeal contamination with *Candida* (4 days)	*H. capsulatum* – detected but not quantified (3 days)	No, started on liposomal amphotericin B empirically based on prior history	Discharged with itraconazole following two-week course of liposomal amphotericin
**75-year-old female with chronic sinus disease who presented with four weeks of fever and was found to have pancytopenia, pulmonary opacities, and pleural effusions.**	Urine *Histoplasma* antigen EIA: 0.3 (7 days)	Blood – no growth Bronchoalveolar lavage – no growth	Bronchoalveolar lavage – No fungal organisms in GMS stain (3 days)	*H. capsulatum* – 25 (5 days)	Yes, started on liposomal amphotericin B	Discharged with liposomal amphotericin B and switched to itraconazole after a ten-day course
**60-year-old male with 40-pack-year history presents with a right lower lobe mass identified a year prior**	Urine *Histoplasma* antigen EIA: <0.2 (6 days)	Right lower lobe tissue culture – no growth	Lung biopsy – Fungal organisms on GMS stain (3 days)	*H. capsulatum* – < 50 (3 days)	Yes, started on liposomal amphotericin B and transitioned to itraconazole after one week of therapy	Discharged to home

mcfDNA, microbial cell-free DNA; NGS, Next-generation sequencing; HIV, Human immunodeficiency virus; AIDS, Acquired immunodeficiency syndrome; HLH, Hemophagocytic lymphohistiocytosis; EIA, Enzyme immunoassay; MPM, Molecules per microliter; GMS, Grocott’s methylamine silver.

All patients had urine *Histoplasma* antigen tests; only four had antigen levels above the assay detection thresholds. One had an immunodiffusion assay which had a positive M band, and one had a negative cerebrospinal fluid antigen test. Seven had fungal cultures of various tissues including blood, bone marrow, skin, cerebrospinal fluid, bronchoalveolar lavage, and valve tissue, three of these cultures were positive. The same seven patients also had a biopsy of corresponding tissue (bone marrow, skin, aortic valve tissue, bronchoalveolar lavage, and lung tissue), of which six yielded histopathologic evidence of *Histoplasma*. Five patients had quantified mcfDNA NGS concentrations, while three underwent an earlier version of the test that did not report quantified results. Among those with quantified results, the results ranged between 65 and 2,729 MPM.

The TAT for mcfDNA NGS was compared with *Histoplasma* antigen testing, fungal cultures, and tissue biopsy or cytology. Median TAT for mcfDNA NGS was 2.5 days (range 2–5 d), compared with 4.5 days (range 2–8 d) for *Histoplasma* urine antigen, 6.5 days (range 6–13 d) for positive fungal culture, and 2 days (range 1–4 d) for tissue pathology or cytology. Antifungal therapy targeting *Histoplasma* was initiated based on mcfDNA NGS results in five of eight patients (62.5%), following biopsy in two patients, and empirically in one patient with a prior history of histoplasmosis. Six were initially treated with liposomal amphotericin B and transitioned to itraconazole following clinical improvement (median of 14 d of amphotericin B therapy, range 10–21 d), while two were started on itraconazole without a prior course of liposomal amphotericin B. Following transition to itraconazole, six were discharged, one was transferred to a long-term acute care hospital, and one died in the hospital from septic shock.

## Discussion

This case series describes eight patients with *Histoplasma* infections and challenging diagnostic courses. Most underwent invasive tissue sampling procedures to obtain cultures and histologic samples. Serum and blood-based diagnostics were frequently negative, and several patients diagnosed by mcfDNA NGS or *Histoplasma* serology still underwent invasive sampling, either while these tests were pending or after results to confirm the presence of fungi in the suspected infection site and establish the clinical syndrome along with the causative organism.

McfDNA NGS has been recognized along with other molecular assays as a valuable modality for diagnosing histoplasmosis^3^ and previous case studies in children and adults have documented its utility when antigen assays and cultures are unrevealing or histopathology is inconclusive.^4–5^ In this case-series, mcfDNA NGS offered a faster diagnosis and facilitated prompt pathogen-directed antifungals in five of eight patients, particularly in those without easily accessible tissue.

Although cytology results had a slightly shorter TAT than mcfDNA NGS, this metric does not account for delays in tissue acquisition, which can be prolonged by clinical instability or logistical barriers to performing invasive procedures. Such delays can substantially extend the time to diagnosis. The mcfDNA NGS assay is therefore most likely to be useful when sent in tandem with the decision to pursue invasive sampling rather than after the sampling has occurred, though it may still be useful when tissue cultures or biopsies are unrevealing or inconclusive.

This study is limited by its small sample, and the inability to ascertain the test’s sensitivity and specificity for this mycosis. Though every person in this series had a compatible syndrome, a positive test result may not necessarily correlate with an imaging finding and thus invasive testing may still be required after mcfDNA NGS results. Larger cohorts are necessary to determine if mcfDNA matches for this fungus can be attributed to an infectious syndrome and to characterize the false-positive rate. Furthermore, the prohibitive cost of the test compared to more widely available commercial assays should also prompt careful consideration of the need for time-sensitive diagnosis before sending patient sera for analysis, particularly in patients with mild or moderate disease for whom rapid treatment may not be immediately necessary.

*Histoplasma* infections are common throughout the United States and not just in the Mississippi and Ohio River Valleys where it has been traditionally thought to be endemic, with its distribution changing over recent decades.^6^ It is therefore not surprising that the manufacturers of the Karius test have found *Histoplasma* mcfDNA to be the most common among dimorphic fungi in samples received by the largest company performing NGS in the United States.^7^ Larger studies are necessary to determine the optimal timing and role for this assay in identifying the large and growing number of patients for whom prompt diagnosis may translate into better outcomes.

## Table 1. Long description

The table presents data on eight patients with histoplasmosis, detailing their clinical characteristics, laboratory testing results, and outcomes. It includes columns for patient descriptions, Histoplasma antigen tests, fungal cultures, fungal stains, tissue biopsies, organisms detected in mcfDNA NGS and concentration, changes in management, and disposition and outcome. The table has eight rows, each corresponding to a different patient, and multiple columns providing specific test results and clinical details. Notable trends include the detection of H. capsulatum in various tests and the subsequent changes in patient management, such as the start of itraconazole or liposomal amphotericin B treatments.

Navigate back to Table 1..
